# Hepatic Encephalopathy after Transjugular Intrahepatic Portosystemic Shunt in Patients with Recurrent Variceal Hemorrhage

**DOI:** 10.1155/2013/398172

**Published:** 2013-03-28

**Authors:** Popovič Peter, Zore Andrej, Šurlan Popovič Katarina, Garbajs Manca, Skok Pavel

**Affiliations:** ^1^Institute of Radiology, University Medical Centre Ljubljana, Zaloška cesta 7, 1525 Ljubljana, Slovenia; ^2^Department of Ginecology, University Medical Centre Ljubljana, Zaloška cesta 7, 1525 Ljubljana, Slovenia; ^3^Department of Gastroenterology and Endoscopy, University Medical Centre Maribor, University of Maribor, Ljubljanska Ulica 5, 2000 Maribor and Medical Faculty, Slomškov trg 15, 2000 Maribor, Slovenia

## Abstract

*Purpose*. The purpose of this study was to determine the incidence and predictors of hepatic encephalopathy (HE) after transjugular intrahepatic portosystemic shunt (TIPS) and endoscopic therapy (ET) in the elective treatment of recurrent variceal hemorrhage. *Methods*. Seventy patients were treated with elective TIPS and fifty-six patients with ET. Median observation time was 46.28 months in the TIPS group and 42.31 months in the ET group. *Results*. 30 patients (42.8%) developed clinically evident portosystemic encephalopathy in TIPS group and 20 patients (35.6%) in ET group. The difference between the groups was not statistically significant (*P* = 0.542; *χ* ^2^ test). The incidence of new or worsening portosystemic encephalopathy was 24.3% in TIPS group and 10.7% in ET group. Multivariate analysis showed that ET treatment (*P* = 0.031), age of >65 years (*P* = 0.022), pre-existing HE (*P* = 0.045), and Child's class C (*P* = 0.051) values were independent predictors for the occurrence of HE. *Conclusions*. Procedure-related HE is a complication in a minority of patients treated with TIPS or ET. Patients with increased age, preexisting HE, and higher Child-Pugh score should be carefully observed after TIPS procedure because the risk of post-TIPS HE in these patients is higher.

## 1. Introduction

Currently, transjugular intrahepatic portosystemic shunts (TIPS) are used in the treatment of complication of portal hypertension in cirrhotic patients. Bleeding from gastroesophageal varices is the most common life-threatening complication of cirrhosis of the liver. Rebleeding has been prevented by reducing portal venous pressure using *β*-blockers, endoscopic interruption of blood flow through varices by sclerotherapy (ET) or varix ligation (EVT), and by placement of TIPS. One of the main drawbacks of TIPS is the development of hepatic encephalopathy (HE) [1]. HE is clinically classified into three major categories, according to the underlying hepatic condition. Type A occurs in patients with acute liver failure. Type B occurs in patients with portosystemic shunting (TIPS). Type C is related to underlying cirrhosis [2–4]. The incidence of HE following TIPS ranges from 20% to 55% within the first two years [4–6]. It can be episodic or persistent. Episodic HE can lead to repeated hospitalizations, often caused by precipitating factors. However, about 3–7% of patients with the TIPS tend to have recurrent or refractory HE, necessitating shunt occlusion or reduction [3–9].

Prevention of HE is difficult. Riggio et al. found no improvement when lactitol and rifaximin were used as HE prophylaxis [10]. Based on this trial, guidelines do not recommend using nonabsorbable disaccharides or antibiotics prophylactically for preventing post-TIPS HE. The evaluation of the predictors of post TIPS HE could probably help clinicians counsel patients before TIPS and reduce HE. Therefore, the aim of this study was to determine the incidence and predictors of hepatic encephalopathy after elective transjugular intrahepatic portosystemic shunt versus endoscopic therapy (ET) in patients with recurrent variceal hemorrhage.

## 2. Materials and Methods

### 2.1. Patient Selection

Between April 1994 and December 2008, we analysed all patients with liver cirrhosis and portal hypertension admitted to our hospital with an episode of acute gastroesophageal variceal rebleeding. The inclusion criteria were as follows: a Child-Pugh score up to 13, at least three gastroesophageal variceal bleedings, or two bleeding episodes occurring less than one month apart. Exclusion criteria included (a) chronic occlusion of the portal vein, (b) hepatocellular carcinoma or other malignant lesions, (c) acute alcoholic hepatitis, (d) age over 75 years, and (e) chronic portosystemic HE. There were 126 patients enrolled in the study. Of these, 70 patients had elective TIPS and 56 had endoscopic therapy (variceal ligation or variceal sclerotherapy) in combination with nonselective beta-blockers. TIPS patients were included in the study at the time of the procedure, that is, on average 32 days after the last bleeding episode. Endoscopic therapy patients were included in the study on average 30 days after the last bleeding episode. TIPS and ET patients were followed up either until the last control examination in December 2008, until death, or until liver transplantation.

### 2.2. Procedures

The technique of TIPS has been extensively described elsewhere and is only briefly discussed here [5, 11–23]. Prior to elective TIPS, patients were haemodynamically and systemically stable. Under sedation, the hepatic vein is catheterized. Using a Colapinto needle catheter, a tract is created between one of the hepatic veins and an intrahepatic portion of the portal vein. Presence of portal hypertension is confirmed by measurement of portal venous pressure. The tract is then dilated and kept patent by deployment of a bare stent across it or with polytetrafluoroethylene- (PTFE-) covered stent grafts. The left gastric vein was not embolized. Neither a parallel stent, prophylactic anticoagulation, nor antibiotics were used in any patient. Because of the recognized complication of HE, the aim of the procedure in our centre was to decrease portal pressure gradient (PPG) to just <12 mmHg or at least 20% from baseline levels using bare stents or covered stents dilated with 10 or 12 mm balloon according to the reduction achieved in pressure gradient.

In the ET group, endoscopic sclerotherapy treatment was performed via paravariceal and intravariceal injection of a sclerosant. The most commonly used sclerosant was 1% polidocanol. Histoacryl adhesive, used in the same proportion as polidocanol, was injected directly into the varices. Usually, the bleeding varix was injected with 1-2 mL of the solution. Endoscopic variceal ligation consists of the placement of rubber bands on the varices that completely interrupt blood flow into the ligated varix. After acute bleeding, ET or EVL was repeated at two–four week intervals if eradication had not previously been performed. Patients received antibiotic prophylaxis prior to and after ET and Sandostatin (1.2 mg/24 hours for 3–5 days) after the procedure.

### 2.3. Followup

Follow-up observation included clinical assessment, upper gastrointestinal endoscopy, ultrasound (US) examination of the abdomen, and examination of shunt flow using colour duplex ultrasound. A clinical examination was performed in all patients every 3 months for the first two years then every 6 months. Endoscopy was performed every 6 months in all patients, and ET was repeated if rebleeding or reappearance of varices occurred. A baseline colour duplex ultrasound examination of the TIPS is performed within 24 hours of its placement. Shunt patency is reassessed at discharge, and the result serves as a reference value for follow-up examinations. Routine surveillance examinations are performed at three-month intervals for the first year and at six-month intervals for the second and subsequent years. If a patient develops recurrent symptoms, variceal bleeding, and/or ascites, the TIPS is examined by US. All suspected shunt abnormalities were confirmed by venography and portosystemic pressure gradient measurements. 

### 2.4. Evaluation of HE

Hepatic encephalopathy was evaluated by assessment of mental status and by serum level of ammonia. Procedure-related HE was defined as new, clinically significant encephalopathy requiring the initiation of treatment in a patient with no previous history of HE, or worsening of pre-existing clinically significant HE requiring either hospitalization or an increase in therapy.

### 2.5. Statistical Analysis

The results are demonstrated in graphical, tabular, and numerical form as means with standard deviation (±SD). The groups were compared using the *t*-test, *χ* ^2^ test, Wilcoxon test, and Mann-Whitney test. Cumulative survival was analysed by the Kaplan-Meier method. The groups were compared using the Kaplan-Meier method and the log rank test. Statistically significant variables determined by univariate analysis were included in the multivariate analysis. Multivariate analysis using the Cox regression model was performed to determine the influence of independent prognostic factors on occurrence of HE. The differences were considered statistically significant at a *P* value of <0.05. The study protocol was approved by the Medical Ethics Committee at the Ministry of Health of the Republic of Slovenia.

## 3. Results

### 3.1. Clinical Data

Both groups were similar at the time of inclusion into the study regarding sex, age, etiology of liver cirrhosis, Child-Pugh classification, number of previous episodes of variceal bleeding, previous hepatic encephalopathy, previous ascites, and site of bleeding (Table 1). Median observation time was 35.47 ± 19.62 months in the TIPS group and 23.6 ± 9.31 months in the ET group. Following TIPS, liver transplantation was performed in 10 patients and following ET in three patients. 

### 3.2. TIPS Procedure

TIPS was performed under general anaesthesia in 68 patients (97.1%) and under local anaesthesia in two patients. Bare stent (10 or 12 mm in diameter and 60–80 mm in length) was used in 50 patients and covered stent (10 mm in diameter) in 10 patients. The average portal pressure was 29.32 ± 5.93 mmHg (range 20–45) prior to the procedure and 18.67 ± 4.22 mmHg (range 8–30) after it. Average decrease of the portal pressure was 35.9% (range 17–62%).

### 3.3. Hepatic Encephalopathy

30 patients (42.8%) in the TIPS group and 20 patients (35.6%) in the EST group developed clinically evident HE. The difference between the groups was not statistically significant (*P* = 0.542; *χ* ^2^ test). Of the patients included in the analysis (*n* = 126), 25.7% had evidence of HE prior to TIPS insertion and 30.4% prior to ET. The incidence of new or worsening HE was less common in the EST group than in the TIPS group (10.7% versus 24.3%). The difference was not statistically significant (*P* = 0.058; Wilcoxon test). In the majority of post TIPS HE, HE resolved with conservative management (withdrawal of diuretics or psychotropic medication or the commencement of lactulose) (*n* = 10), the use of antimicrobials (*n* = 6), and the treatment of rebleeding (*n* = 1). Multivariate analysis according to the Cox regression model included treatment modality, age, sex, etiology of liver disease, pre-existing HE, Child's class, bilirubin, ALT, albumin levels, and ascites. Further analysis revealed pre-existing HE to be the only significantly predictive variable in determining the subsequent development of HE after TIPS insertion (*P* = 0.04).

### 3.4. Rebleeding

A significantly higher number of patients with rebleeding episodes were observed in the ET group (36 patients, 64.3%) than in the TIPS group (15 patients, 21.4%; *P* = 0.001; *χ* ^2^ test). After two years 7 (9.8%) patients rebleed in TIPS group and 31 (55.4%) patients in ET group. The probability of being free of variceal rebleeding during the first and fourth years of followup was significantly higher in TIPS than in ET patients (92.4% versus 59.5%; 82.6% versus 40.6%; *P* = 0.03).

### 3.5. Survival

Cumulative survival in the TIPS group was 83% after one year and 73.5% after four years. In the ET group, survival was 69.8% after one year and 39.8% after four years. A statistically significant difference was established between the two groups (*P* = 0.013 log-rank; *P* = 0.024 Wilcoxon test). In the TIPS group, the main cause of death was liver failure, whereas in the ET group it was variceal rebleeding.

## 4. Discussion

In 13 randomized studies that compared TIPS to ET, the authors report that failure to improve survival and increased incidence of HE delimit the use of TIPS as the method of choice for prevention of recurrent variceal bleeding [11–23]. The incidence of new or worsening HE following TIPS is 20–54% [6, 11–23]. The incidence and severity of HE are higher during the first month after a TIPS procedure and decrease progressively because the diameter of the shunt tends to decrease spontaneously. This theory is confirmed by the increase in portosystemic gradient index and reduction in ammonia levels during the follow-up period, especially in patients treated with bare stent [3]. The discrepancy of the results reported is possibly attributable to different definitions of HE associated with TIPS, as well as to the heterogeneity of the subjects studied, differences in followup and HE evaluation methods. We did not observe statistically significant differences between groups concerning the rate of HE episodes following TIPS and ET and the incidence of postprocedure HE associated with TIPS and ET. In comparable studies, the authors report higher HE incidence in patients treated by TIPS than in patients undergoing ET: 29%–54% versus 7%–26%, respectively [11–23]. Two studies documented similar results for TIPS and ET patients (22.7% versus 25% and 50% versus 44%) [15, 19]. In three studies, procedure-related HE was defined as new, clinically significant encephalopathy requiring initiation of treatment in a patient with no previous history of HE, or worsening of preexisting clinically significant HE requiring either hospitalization or an increase in therapeutic approach. In four studies, HE associated with a procedure was defined as a spontaneous episode of clinically verified HE. 

Similar results in our study in both groups seem to be due to similar clinical characteristics of the patients prior to the procedure: the same number of patients with severe liver disease, same number of patients with pre-existing HE and nonalcoholic liver disease, and similar age of patients in both groups. Lower rates of HE observed in the elective TIPS group are also attributable to haemodynamic stability and compensated liver function in these patients. HE occurred mostly as a result of rebleeding, alcoholic intake, or aggressive diuretic therapy without requiring hospital admission. The rebleeding rate in our TIPS patients at two years is lower than the rate in other comparable studies (9.8% versus 19%; range 9%–41%) [11–23]. The difference may be ascribed to patient selection, as well as to regular US monitoring of shunt patency and timely and effective additional interventions.

The incidence of HE in our study was similar to that reported in the literature with covered stents [24–28]. Post TIPS HE is anticipated to be higher with a wider shunt lumen. Thus, its frequency and severity would be expected to be higher with covered stents, as its diameter remains unchanged over a long period of time, unlike bare stents, which show progressive reduction of the shunt diameter from intimal hyperplasia. Interestingly, not only has the incidence of HE been found to be similar with either device, but also some studies have in fact shown a lower frequency of HE with covered stent [28, 29]. However, a recent randomized trial by Riggio et al. comparing 8 mm and 10 mm shunts clearly showed no difference in HE rates [30]. The authors additionally showed the 8 mm shunts to be ineffective in portal decompression and hence do not recommend their use over the 10 mm shunts, which is consistent with the results of our study.

Another probable reason for lower HE incidence following TIPS was most likely the smaller diameter of the shunt in our study (10–12 mm). Similarly, Sarfeh et al. [31] noted that HE incidence, which was 39% for shunts 16–20 mm in diameter, fell to 9% for portocaval H-shunts measuring only 8 mm. Johansen [32], who used smaller portocaval shunts (10–12 mm) in Child class A and B patients, reported an incidence of HE of only 6%. 

Previous studies showed that predictive factors of the development of post TIPS HE were non-alcoholic causes of cirrhosis, hypoalbuminemia, older age, Child-Pugh class B or C, previous episodes of HE, and a high degree of reduction of the portosystemic gradient [3, 9]. Multivariate regression analysis of our study identified that pre-existing HE was the significant independent predictor of HE during followup. 

Our study showed that procedure-related HE is a complication in a minority of patients treated by TIPS or ET. In our series and in those reported in the literature, HE occurred mostly as a result of alcoholic intake, rebleeding, infection, dehydration, or aggressive diuretic therapy, and it disappeared with proper diet and therapy without requiring hospital admission. Accurate estimates of the incidence and severity of HE after TIPS placement are precluded by heterogeneity across studies, different definitions of HE occurring after TIPS, and by a number of open questions concerning ammonium metabolism, brain adaptation to neurotoxins produced within the gastrointestinal tract, and changes in astrocyte metabolism.

## Figures and Tables

**Table 1:** Demographic and clinical characteristics of the patiens.

	TIPS (*n* = 70)	ET (*n* = 56)	*P*
Sex (*n*)			
Male	45	35	*P* = 0.491
Female	25	21	
Mean age ± SD	53.56 ± 11.6	57.57 ± 11.7	*P* = 0.524
Etiology of liver cirrhosis (*n*)			
Alcohol	49 (70%)	38 (67.9%)	*P* = 0.473
Postinflammatory	21 (30%)	18 (32.1%)	
Child class (*n*) (mean ± SD)			
A	15 (21.4%)	8 (14.3%)	
B	41 (58.6%)	31 (55.4%)	*P* = 0.319
C	14 (20%)	17 (30.4%)	
Child score (mean ± SD)	8.68 ± 1.9	8.43 ± 1.7	*P* = 0.509
Bleedings per patient (mean ± SD)	3.46 ± 1.1	3.36 ± 1.1	*P* = 0.618
Encephalopathy (*n*)	18 (25.7%)	17 (30.4%)	*P* = 0.352
Ascites (*n*)	39 (55.7%)	36 (64.3%)	*P* = 0.455
Site of bleeding (*n*)			
Oesophagus	46 (65.7%)	41 (73.2%)	
Gastric	7 (10%)	6 (10.7%)	*P* = 0.526
Oesophagus/gastric	17 (24.3%)	9 (16.1%)	
